# Intracardiac and Cerebral Thrombosis Complicated With Mycoplasma Pneumonia

**DOI:** 10.7759/cureus.60563

**Published:** 2024-05-18

**Authors:** Weisheng Huang, Jingxia Hao, Yingqian Zhang

**Affiliations:** 1 Medicine, Hebei Medical University, Shijiazhuang, CHN; 2 Pediatric Cardiovascular Disease, Children's Hospital of Hebei Province, Shijiazhuang, CHN; 3 Pulmonology, Children's Hospital of Hebei Province, Shijiazhuang, CHN

**Keywords:** young child, antibiotic, anticoagulant therapy, thrombosis, mycoplasma pneumoniae infection

## Abstract

A seven-year-old girl developed multiposition thrombosis after fever and respiratory symptoms. Chest computed tomography (CT) scan demonstrated bilateral infiltrates, consolidation of the right lower lobe, and pleural effusion in the right lung field. Brain magnetic resonance imaging (MRI) showed multiple abnormal signals in the brain with limited diffusion, and cerebral infarction could not be excluded. Echocardiography revealed hypoechoic mitral valve tips, which are likely to be suspected as vegetation. *Mycoplasma pneumoniae* infection was clarified by a four-fold increase in IgG antibodies to *M. pneumoniae* sera. D-dimer levels were elevated increasingly.

We found and reported this rare pediatric case of an *M. pneumoniae*-induced severe pneumonia complicated with intracardiac and cerebral thrombosis. We investigate the clinical characteristics, diagnosis, and treatment of refractory mycoplasma pneumonia complicated with intracardiac and cerebral thrombosis in children.

## Introduction

*Mycoplasma pneumoniae* infection is a common pathogen of community-acquired pneumonia in children from the period when admitted to primary school to one during the adolescence period. *M. pneumoniae* infection can cause pulmonary symptoms, meanwhile extrapulmonary complications of *M. pneumoniae* infection, including the skin, mucous membranes, blood system, nervous system, digestive system, and cardiovascular system, are not rare [1,2]. According to the reported cases in China, CAP is prevalent in children and teenagers. In recent several years, many severe or even fatal cases of mycoplasma pneumonia have been reported in Asia, particularly in Eastern Asia.

Of the human diseases that have proven to be due to mycoplasmas, pneumonia caused by *M. pneumoniae* is by far the most clinically important [1,3]. Patients with or without respiratory symptoms can also have other related symptoms, such as skin disease and heart disease [3]. In recent years, mycoplasma pneumonia has caused blood hypercoagulability and leads to thrombosis, such as pulmonary embolism, cerebral infarction, middle retinal artery obstruction, and deep vein thrombosis.

## Case presentation

The patient, a seven-year-old girl, was admitted to the hospital because of a fever for 13 days. There is no exposure to any pathogen. The girl had paroxysmal cough with sputum without obvious cause for two weeks, with a maximum body temperature of 40.3°C. Laboratory examination showed a white blood cell (WBC) count of 8.35×10^9^/L, comprising 87.8% neutrophils, 8.5% lymphocytes, and 3.7% monocytes, and platelets (PLT) count of 8.35×10^9^/L (first column in Table 1). Chest CT scan demonstrated right lobar pneumonia. Although she had taken oral and intravenous azithromycin prescribed for a total of five days before being admitted to our hospital, her symptoms did not show improvement.

**Table 1:** Blood count and CRP HGB, hemoglobin; PLT, platelet; WBC, white blood cell; NE%: the percentage of neutrophils in white blood cells; LY%: the percentage of lymphocytes in white blood cells; CRP, C-reaction protein

	One week before hospitalization	Five days before hospitalization	One day before hospitalization	First day during hospitalization	Third day during hospitalization	Normal
HGB (g/L)	144	147	129	110	109	110-160
PLT (×10^9^/L）	192	129	77	33	56	100-300
WBC (×10^9^/L）	8.35	10.3	14.4	10.9	14.3	4-10
NE%	87.8	82.5	87.8	88.5	87.2	50-70
LY%	8.5	15.3	8.5	4.8	6.4	20-40
CRP (mg/L）		32.2	32.31	30.59	5.61	0-6

**Figure 1:**
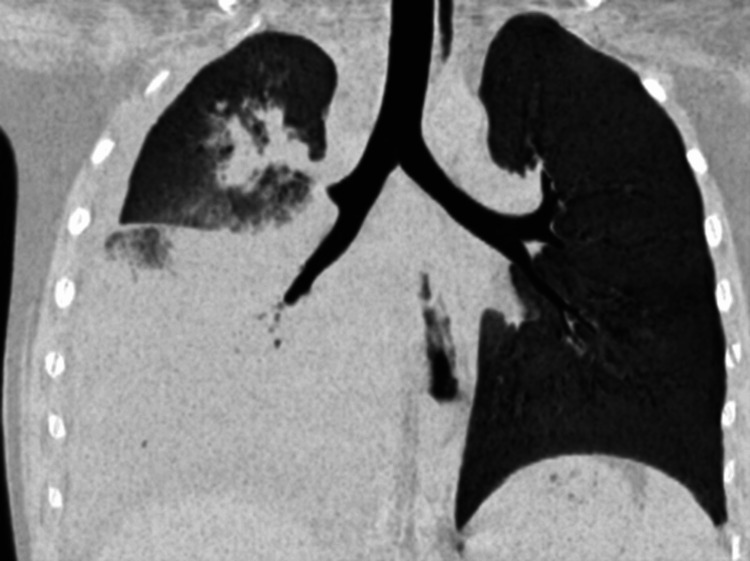
Chest CT scan demonstrates bilateral infiltrates, consolidation of the lower lobe of the right lung and right pleural effusion. CT, computed tomography

For further treatment, the patient was admitted to Hebei Children’s Hospital. Laboratory examination showed an HGB of 147 g/L, WBC of 10.3×10^9^/L, comprising 15.3% lymphocyte, 82.5% neutrophil, PLT of 128×10^9^/L, C-reaction protein (CRP) of 32.2 mg/L, and serum amyloid A (SAA) of >500 mg/L (second column in Table 1). Antibody Immunoglobin M (IgM) to influenza B virus is positive. Chest computed tomography (CT) scan conducted before admission demonstrates the presence of bilateral infiltrates, consolidation of the lower lobe of the right lung, and right pleural effusion (Figure 1). After the treatment of azithromycin, methylprednisolone, and peramivir, she was admitted to the respiratory department with the initial diagnosis of "pneumoniae, lung consolidation, and pleural effusion."

On admission, she was clear and mentally good. Vital signs showed a body temperature of 38.1℃, respiratory rate of 30 breaths/min, and heart rate of 122 beats/min.

**Figure 2:**
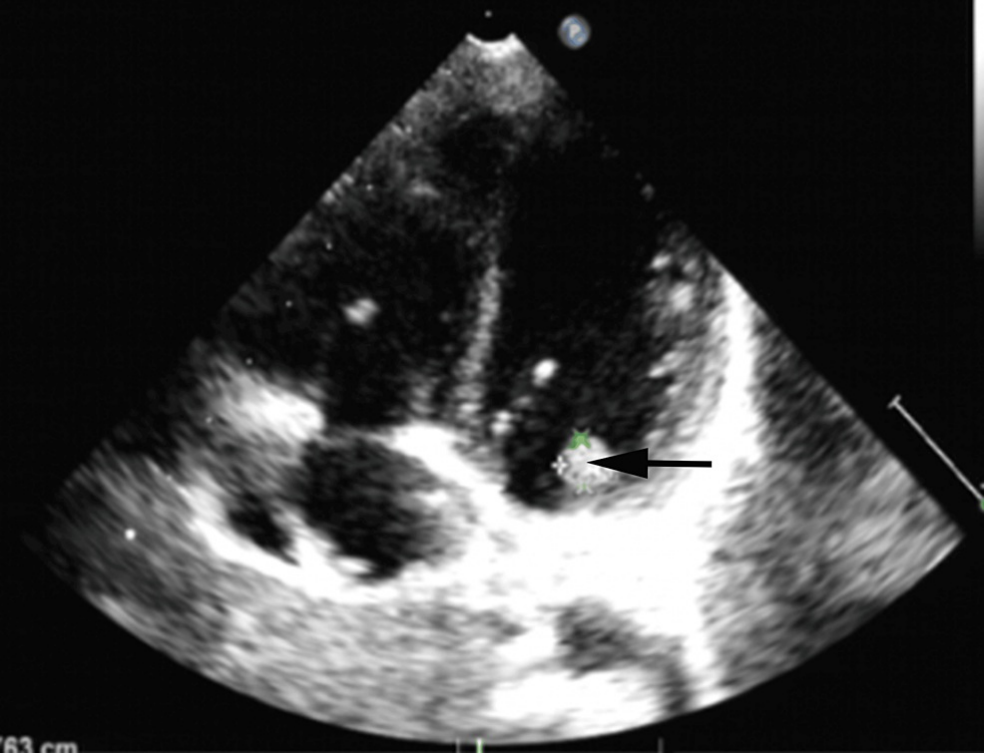
Echocardiography demonstrates hypoechoic mitral valve cusp (black arrow)

Coagulation function revealed an elevated PT of 13.8 sec, international normalized ratio (INR) of 1.21, and D-dimer of 8.17 mg/L FEU (first column in Table 2). Serum protein tests show a TP of 51.7 g/L and albumin (Alb) of 32 g/L. Liver function and cardiac enzymes show an ALT of 102 U/L and AST of 13 0U/L. Electrolytes tests show K^+^ of 3.36 mmol/L, sodium Na^+^ of 127.9 mmol/L, Cl^‑^ of 93.3 mmol/L, HCO_3_^- ^ of 22.3 mmol/ L (first column in Table 3). Serum antibody titers to *M. pneumoniae* were 1:1280. Thoracic ultrasound demonstrates right pleural effusion and partial consolidation of the right lung. ECG shows sinus tachycardia. Echocardiography demonstrates hypoechoic mitral valve cusp, which is likely to be suspected as vegetation, small regurgitation of the mitral and tricuspid valves, and pericardial effusion (Figure 2). Neither the upper extremities' arteriovenous ultrasound nor the lower one suggests any obvious abnormalities, suggesting no thrombosis exists.

**Table 2:** Coagulation function PT, prothrombin time; INR, international normalized ratio; TT, thrombin time; APTT, activated partial thromboplastin time

	First day during hospitalization	Third day during hospitalization	Normal
PT (sec)	11.7	11.2	9.8-12.1
INR	1.02	0.96	0.80-1.20
TT (sec）	17.3	17.0	14.0-21.0
APTT (sec）	25.9	26.6	25.0-31.0
D-dimer (mg/L FEU）	8.17	12.74	0-0.5

**Table 3:** Electrolyte, liver function, and cardiac enzyme TP, total protein; Alb, albumin; ALT, alanine aminotransferase; AST, aspartate aminotransferase; LDH, lactate dehydrogenase; HBDH, hydroxybutyrate-dehydrogenase; CK, creatine kinase; CK-MB: a type of CK, it reveals the function of the heart

	On the day of hospitalization	First day during hospitalization	Normal
Na^+ ^(mmol/L)	127.9	133.1	135-145
K^+ ^(mmol/L）	3.36	3.09	3.7-5.2
Cl^- ^(mmol/L）	93.3	96.2	98-110
HCO_3_^- ^(mmol/L）	22.3	24.7	23-29
TP (g/L)	51.7	51.3	65-84
Alb (g/L)	21	30.8	39-54
ALT (U/L)	102	105	7-30
AST (U/L)	130	92	14-44
LDH (U/L)	1929	1828	109-245
HBDH (U/L)	1579	1613	72-182
CK (U/L）	590	200	40-200
CK-MB (μg/L)	3.59	<3	<5

**Figure 3:**
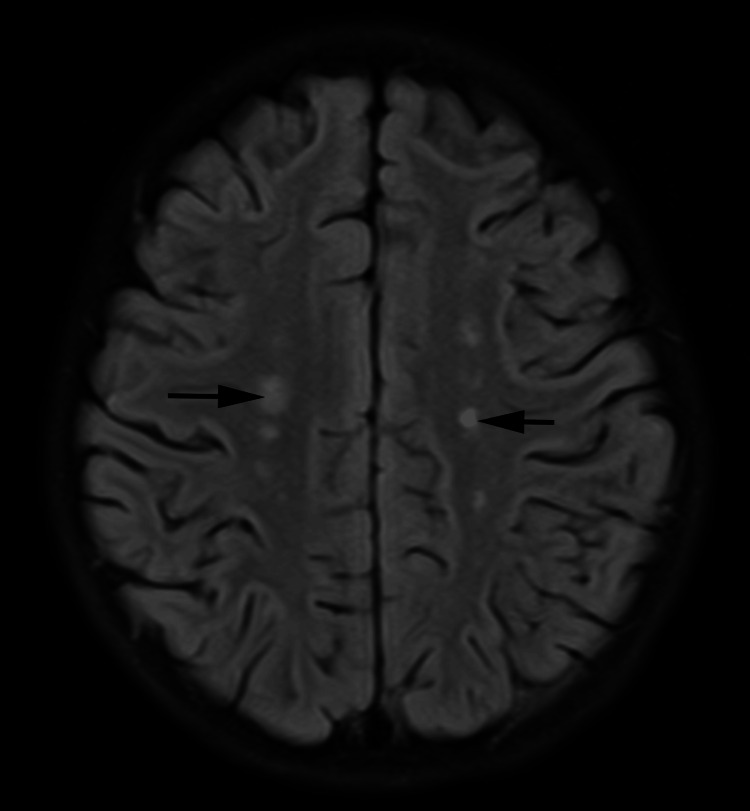
Brain MRI showed multiple abnormal signals in the brain with limited diffusion (black arrow) MRI, magnetic resonance imaging

The patient was diagnosed with pneumoniae, suggestive of *M. pneumoniae* origin associated with intracardiac and cerebral thrombosis. Antibiotics of azithromycin at doses of 10 mg/(kg·d), peramivir at doses of 10 mg/(kg·d), norvancomycin at doses of 24 mg/(kg·d), cefotaxime with sulbactam at a dose of 15 mg/(kg·d) were administered for one day and methylprednisolone for one day. After being transferred to the Department of Cardiology, antibiotics of desvancomycin at doses of 24 mg/(kg·d), oseltamivir at doses of 10 mg/(kg·d), doxycycline at doses of 0.1 g/d are given. Laboratory examination after treatment shows a HGB of 109 g/L, WBC of 14.3×10^9^/L, consisting of 6.4% lymphocyte, 87.2% neutrophil, PLT of 56×10^9^/ L, CRP of 5.61 mg/L (the last column in Table 1). D-dimer decreases to 12.74 mg/LFEU (second column in Table 2). Electrolyte exams show a Na^+^ of 136.3 mmol/L, K^+^ of 3.80 mmol/L, and Cl^-^ of 100.0 mmol/L. Serum protein shows a TP of 51.3 g/L and Alb of 30.8 g/L. Liver function and cardiac enzyme show ALT of 105 U/L and AST of 92 U/L (second column in Table 3). Analysis of auto-antibodies including antinuclear antibodies, anticytoplasmic antibodies, anti-DNA antibodies, and rheumatoid factors were all negative. Analysis of the patient's CSF showed a total cell of 1908×10^9^/L, WBC count of 8×10^9^/μL, and protein concentration of 0.19 g/L. T1-weighted and T2-weighted MRI shows abnormally high signal intensity in bilateral semiovoid center, radial crown, paraventricular, frontal-occipital-parietal cortical area, and subcortex, cerebellar hemispheres. The fluid-attenuated inversion recovery (FLAIR) sequence provides high signal intensity, and diffusion-weighted imaging (DWI) demonstrates high signal in the above lesions and occipital cortex and subcortex (Figure 3). While MRA presents a good view, as for the outcome of MRV the left transverse sinus of the cranial MRV is locally faintly apparent. Notably, there is no relation between abnormal signals and faintly apparent sinus, because they derive from different regions. After the combination of antibiotics, anti-coagulants, and anti-thrombotic therapy, the girl did not cough any longer, without neither fever nor thrombosis.

## Discussion

*M. pneumoniae* is a common cause of CAP in children and adults, accounting for nearly 20% of all CAP in middle and high school children and up to 50% of CAP in college students and military recruits [1]. According to the reported cases in China, CAP is prevalent in children and teenagers. In recent years, many severe or even fatal cases of mycoplasma pneumonia have been reported in Asia, particularly in Eastern Asia.

Of the human diseases that have proven to be due to mycoplasmas, pneumonia caused by *M. pneumoniae* is by far the most clinically important [1,3]. Patients with or without respiratory symptoms can also have other related symptoms, such as skin disease, and heart disease [3].

Echocardiography demonstrates hypoechoic mitral valve cusp in the case, which can be attributed to either thrombosis or infective endocarditis (IE). Owing to the previous infection history, the best explanation should be IE. IE is defined by infection of a native or prosthetic heart valve, the endothelium, and the endocardial surface of the heart [4]. The causes and microbiology of the disease have changed a lot in recent decades. *Staphylococcus aureus* is now the most prevalent cause of IE in most studies at 26.6% of all cases, followed by viridians group streptococci at 18.7%, other streptococci at 17.5%, and enterococci at 10.5%. These organisms together account for 80-90% of all cases of endocarditis [4]. As for the left causes, although there is evidence that *M. pneumoniae* can exactly lead to IE, IE is the rarest symptom due to M. pneumoniae. What’s more, after the anti-coagulation therapy, the D-dimer lever is on the decline. Thus, as far as we are concerned, the best explanation for the echocardiograph is thrombosis. 

Thromboembolism associated with *M. pneumoniae* poses a threat to pediatricians. In previously healthy children, lobar pneumonia or pleural effusion caused by mycoplasma infection is associated with elevated D-dimer and is prone to thrombosis [5]. The time of thrombosis in *M. pneumoniae* varies greatly, with a course of 5-31 days, and 35% of patients have no clinical manifestations related to thrombosis. Previous data showed that thrombosis in the brain and abdomen can occur early, at five days after disease onset [6].

Though not completely understood, three mechanisms have been proposed in order to support the likely pathology of cerebral arterial occlusion. The first is the direct invasion of pathogenic microorganisms after *M. pneumoniae* infection. It releases toxins, causing endothelial damage and oxidative damage in the body, and releases inflammatory cytokines, such as TNF-α, IL-6, and IFN-gamma, causing endothelial dysfunction [7-11]. Second, *M. pneumoniae* antigen has common antigens with multiple organs of the human body, such as the heart, brain, and kidney, and it can produce autoantibodies and form immune complexes, resulting in the occurrence of a variety of extrapulmonary complications after infection [12,13]. What's more, vascular occlusion can cause blood flow obstruction due to vasculitis. Third, hepatocyte damage caused by *M. pneumoniae* infection, hypoxia, and immune injury can increase endogenous coagulation factor synthesis, while the second synthesis of critical anticoagulation factors such as AT-III, protein C, and protein S is reduced. The last plausible hypothesis is the absence of anticoagulant protein. AT-III, protein C, and protein S are anticoagulant proteins synthesized by the liver. AT-III and protein C bind to activated coagulation factors to inactivate coagulation factors and maintain the dynamic balance between coagulation and anticoagulation, and protein S is a cofactor of activated protein C [14,15]. 

Cases of elevated coagulation factors and abnormal anticoagulation proteins have also been reported in MP infection combined with embolism [7,10]. In this case, the D-dimer levels are elevated increasingly. Fortunately, the patient did not owe any manifestation of cerebral and intracardiac thrombosis.

In addition, the CK, as well as its subtype CK-MB, is elevated. *M. pneumoniae* infection has something to do with the abnormal cardiac enzyme spectrum. The Ca^2+^-dependent cytotoxic nuclease produced by *M. pneumoniae* can lead to apoptotic-like programmed cell death in the host [11].

Thrombosis associated with *M. pneumoniae* can manifest differently. There are currently only a few cases of mycoplasma infection with intracardiac and cerebral thrombosis reported in the literature. Fu et al. reported pulmonary in five cases, cerebral in two cases, pulmonary and cardiac in two cases, and lower extremity artery and cardiac thrombosis in one case [12]. Intracardiac thrombosis can be the only type of thrombosis present in a patient, and it can be asymptomatic. It often occurs in the right heart chamber and close to the tricuspid valve [16]. In our case, vegetation attached to the left ventricular aspects of the valve cups was found accidentally by echocardiography as the first manifestation.

Severe mycoplasma pneumonia with pulmonary consolidation was the most strongly associated risk factor for thrombosis. Among those cases reported from the embolism group, most cases are extracted from pulmonary embolism, whereas ventricle or cerebral embolism occurs rarely [17].

The treatment of thrombosis-associated *M. pneumoniae* infection mainly includes anti-coagulation therapy, antithrombotic therapy, and surgical therapy. There is currently no standard regimen for antithrombotic therapy. The anticoagulants currently in most widespread use in children include unfractionated heparin, LMWH, Vitamin K, and primarily warfarin [18,19]. The commonly used antithrombotic drugs are urokinase, streptokinase, and recombinant tissue plasminogen activator, and the treatment window is within three hours of onset, and this procedure should be finished within six hours.

## Conclusions

We report a case of embolism with mycoplasma pneumonia. Thorough evaluation of possible predisposing factors for thrombosis should always be conducted in children and close attention is required to determine the potential systemic hypercoagulable status associated with *M. pneumoniae*-associated thrombosis.
